# Installation of LYRM proteins in early eukaryotes to regulate the metabolic capacity of the emerging mitochondrion

**DOI:** 10.1098/rsob.240021

**Published:** 2024-05-22

**Authors:** Vít Dohnálek, Pavel Doležal

**Affiliations:** ^1^ Department of Parasitology, Faculty of Science, Charles University, BIOCEV, Vestec 252 50, Czech Republic

**Keywords:** LYRM proteins, mitochondrial evolution, LECA, acyl-ACP

## Abstract

Core mitochondrial processes such as the electron transport chain, protein translation and the formation of Fe–S clusters (ISC) are of prokaryotic origin and were present in the bacterial ancestor of mitochondria. In animal and fungal models, a family of small Leu-Tyr-Arg motif-containing proteins (LYRMs) uniformly regulates the function of mitochondrial complexes involved in these processes. The action of LYRMs is contingent upon their binding to the acylated form of acyl carrier protein (ACP). This study demonstrates that LYRMs are structurally and evolutionarily related proteins characterized by a core triplet of α-helices. Their widespread distribution across eukaryotes suggests that 12 specialized LYRMs were likely present in the last eukaryotic common ancestor to regulate the assembly and folding of the subunits that are conserved in bacteria but that lack LYRM homologues. The secondary reduction of mitochondria to anoxic environments has rendered the function of LYRMs and their interaction with acylated ACP dispensable. Consequently, these findings strongly suggest that early eukaryotes installed LYRMs in aerobic mitochondria as orchestrated switches, essential for regulating core metabolism and ATP production.

## 1. Introduction

**Figure 1.**
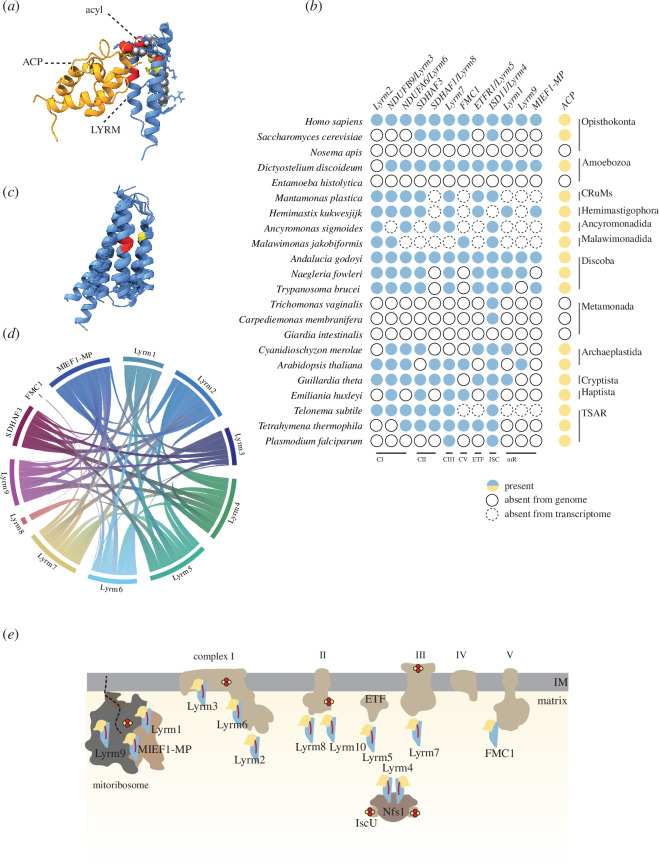
Eukaryotes have 12 different LYRM proteins that regulate the activity of the core mitochondrial processes. (*a*) The structure of the prototypical LYRM protein, human Lyrm4 (Isd11) (light blue), its triple α-helix accommodates acyl chain (grey) bound to ACP (orange), signature LYR motif with the F residue is depicted by red and yellow, respectively, on the α-helices of the Lyrm4. (*b*) Distribution of LYRM subfamilies across selected representatives of eukaryotic supergroups. CI–CV, respiratory complexes I–V; ETF, electron-transferring flavoprotein; ISC, iron sulfur cluster pathway; mR, mitoribosome. (*c*) The uniform structure of the LYRM proteins is demonstrated by the structural superposition of the central domain of all 12 human members of the protein family. (*d*) Chord diagram representing mutual structural similarity among different LYRMs as identified by FoldSeek. (*e*) Schematic representation of the function of LYRM proteins in the activation/assembly of components of key mitochondrial processes. Fe–S clusters are depicted as red and yellow circles.

Leu-Tyr-Arg motif-containing proteins (LYRMs), small mitochondrial proteins characterized by their N-terminal Leu-Tyr-Arg (LYR) motif, have recently gained recognition for their impact on critical mitochondrial functions, such as oxidative phosphorylation (OXPHOS), protein translation and the Fe–S cluster assembly [1–5]. Depending on the functional context, LYRMs are thought to function as assembly factors, activators or accessory subunits that facilitate the activity or folding of their target proteins. However, despite this versatility, a unifying mechanism underlying their different roles remains unknown. Encoded in the nucleus, LYRMs are synthesized on cytosolic ribosomes and subsequently imported into mitochondria. Within the mitochondria, they engage in bipartite interaction with their targets, typically a subunit of larger protein complexes, and with an acyl carrier protein (ACP) bound to an acyl chain [6]. Some LYRMs have been demonstrated to consist of three α-helices that form a hydrophobic tunnel, accommodating the acyl moiety of acylated ACP [7,8] (figure 1*a*). Their involvement in the central mitochondrial functions means that defects in LYRMs have been linked to severe diseases, such as encephalopathy (Lyrm7), insulin resistance (Lyrm1) and various mitochondrial disorders (Lyrm3) [9–11]. Importantly, these phenotypes can be replicated in other cellular systems [12] underscoring their conserved fundamental roles in eukaryotes.

In this study, we aim to elucidate the evolutionary trajectory of LYRMs in eukaryotes, expanding the analyses of their distribution and structural features beyond the commonly studied animal and fungal models. Our findings demonstrate that LYRMs not only share the LYR motif but are also structurally related homologues, belonging to a single protein superfamily. We establish that a complete set of 12 distinct LYRMs was already in place in the last eukaryotic common ancestor (LECA). However, these LYRMs were absent in the prokaryotic ancestor of mitochondria and the host cell. Importantly, homologous prokaryotic complexes involved in Fe–S cluster assembly, protein translation and respiration do not require LYRMs for their function. Nevertheless, in theory, the prokaryotic complexes could be bound by LYRMs if co-present in a cell. This suggests that LYRMs were installed into the early mitochondrion to function as a vital metabolic switch to control its metabolic activity. In contrast, the adaptation of mitochondria to anoxic environments appears to have resulted in the secondary loss of LYRM-mediated regulation in eukaryotes.

## 2. Results

### 2.1. LYRMs are conserved across eukaryotic diversity but are lost in anaerobes

Given the minimal knowledge of the LYRMs outside the main experimental models, we set up bioinformatic searches to map their abundance across the eukaryotic tree of life. Hidden Markov model (HMM)-based searches against the EukProt [13] and UniProtKB [14] databases were conducted using both the seed alignments from the Pfam database and our custom multiple sequence alignments (MSAs). The analogous approach was taken to detect the presence of ACP in eukaryotes. The main aim was to identify the diversity of LYRMs in eukaryotes and the possible absence of both LYRMs and ACP in some eukaryotic lineages.

Of the broad dataset, 22 eukaryotic species were selected to represent the diversity of eukaryotes (figure 1*b*). While the searches revealed that both, the LYRMs and ACP are ubiquitous in eukaryotes, a closer examination revealed the number of LYRMs varied dramatically in different eukaryotic lineages (electronic supplementary material, table S1 and figure 1*b*). A common trend was observed among organisms that have replaced their ‘classical’ aerobic mitochondria with mitochondria-related organelles (MROs). These organisms typically have fewer LYRM proteins, primarily owing to the loss of OXPHOS and the mitoribosome [15], which are the main interaction sites for many LYRMs. In our analysis, three major clades were identified that lack both LYRMs and ACP. These include the entamoebas, notably the human parasite *Entamoeba histolytica*; the majority of Metamonada from the Fornicata clade, such as *Giardia intestinalis* and several species of Microsporidia. (electronic supplementary material, table S1 and figure 1*b*). These eukaryotes commonly inhabit anoxic environments or in the case of Microsporidia, live as intracellular parasites. Their cellular functions converged to the formation of highly reduced MROs known as mitosomes. The complete absence of LYRMs is unexpected, especially considering the established role of microsporidian and metamonad mitosomes in the Fe–S cluster synthesis [16–18], in which Lyrm4 in complex with ACP should play an essential role (see below) [19].

Interestingly, a significantly reduced set of LYRMs was identified in the Apicomplexa, a group of obligatory parasites, which include organisms such as *Plasmodium*, *Toxoplasma* or *Theileria*. While these are not anaerobes, their mitochondria exhibit unique metabolic adaptations, such as the loss of pyruvate dehydrogenase complex, respiratory complex I and the reduction of subunit number of other complexes [20,21].

### 2.2. Different LYRMs belong to a single superfamily of proteins

For the evolutionary analysis, it was crucial to determine whether the similarity among different LYRMs was confined to the amino acid LYR motif, possibly as a result of convergent evolution as suggested by some previous studies [22], or if these proteins constituted a family of with common ancestry. Given the typically short sequences of LYRMs (usually around hundreds of amino acids), creating a single dataset for all LYRMs for reliable phylogenetic reconstruction was not feasible. In addition, initial analysis indicated that even clear functional orthologues of individual LYRMs share only limited primary sequence identity, which posed a significant challenge for pairwise sequence comparison, for example, human Lyrm7 shares 28% sequence identity with orthologue from yeast only 14% identity with *Arabidopsis thaliana* Lyrm7. To address this, we utilized HMM- or structure-based approaches known for their enhanced sensitivity in detecting homology [23,24].

First, the protein sequences from the selected 22 eukaryotes were analysed using an InterProScan (Paysan-Lafosse *et al*., 2023) which classifies protein sequences according to the protein families. For sequences where InterProScan proved insufficient, we employed the HHblits algorithm, based on HMM profiles comparison (Zimmermann *et al*., 2018). Here, the proteins were classified according to the top hit that returned in the search. Using this approach, we could assign each of the 129 LYRMs to one of the 12 subfamilies. However, it became apparent during classification that single queries often cross-recognized multiple LYRM subfamilies.

Thus, to further test the homology among the twelve different LYRMs we took advantage of structural models predicted by AlphaFold2 [25]. We began aligning structural models of all twelve human LYRMs using DeepAlign [26], revealing a high degree of structural similarity despite their participation in distinct mitochondrial interactions (figure 1*c*). Next, we used these models as queries for FoldSeek [27] searches against the AlphaFold structure database [25]. Specifically, the structure of a single LYRM protein was used as a query against all protein structures of the same species. This approach was again first applied to human LYRMs (figure 1*d*). Although mutual structural similarity among LYRMs varied, at least five different LYRMs were identifiable for the most diverged proteins like FMC1 and Lyrm8 (electronic supplementary material, table S2). However, most proteins demonstrated clear structural homology with 8 or 9 other LYRMs. This pattern of structural similarity across different LYRMs was also observed in other unrelated eukaryotic species, such as *Arabidopsis thaliana* and *Dictyostelium discoideum* (electronic supplementary material, figure S1). According to the most parsimonious explanation, this shared structural similarity among various LYRMs supports their affiliation to single protein superfamily. Together with the sensitivity of protein-specific HMMs to recognize the same LYRMs across the eukaryotic spectrum, these results strongly suggest a common evolutionary origin for all LYRM proteins, as opposed to an independent convergence of proteins sharing the LYR amino acid motif suggested earlier [22].

### 2.3. Diversity and functional roles of LYRM protein subfamilies in eukaryotic organisms

We further expanded our study by integrating the classification the LYRMs across eukaryotic diversity with available functional data (figure 1*b,e*). Lyrm4 emerges as the most abundantly represented LYRM across eukaryotic diversity, being nearly universally present. Originally described as Isd11, Lyrm4 with bound acyl-ACP is at the core of mitochondrial Fe–S cluster assembly through the ISC pathway [28]. In this process, it interacts with cysteine desulfurase Nfs1, which provides sulfur ions for the synthetic reaction [5]. The resulting trimer Nfs1–Isd11–ACP dimerizes to form an active complex [19]. Notably, in anaerobic eukaryotes where all but one LYRM have been lost, Lyrm4 is consistently the one retained [29]. This aligns well with the idea that the ISC pathway holds fundamental evolutionary importance for all eukaryotes and has persisted even in MROs, which have lost all other metabolic reactions including ATP production [30].

The next most prevalent is Lyrm7. The protein functions as a chaperone affecting the stability of Rieske Fe–S protein before its insertion into the complex III [31,32]. It also assists the transfer of Fe–S cluster from the ISC pathway component, IscU, via the interaction with the Fe–S transfer chaperon complex [33].

LYRMs also interact with other OXPHOS complexes, except for Complex IV (cytochrome c oxidase). In the case of Complex I, Lyrm2/Lyrm3/Lyrm6 regulates the assembly and the activity of the complex. While the latter two are themselves structural subunits of Complex I [34,35], Lyrm2 was shown to affect the complex assembly only indirectly [3,36]. These LYRMs are present in most eukaryotic lineages, but they are absent in *Saccharomyces cerevisiae*, which aligns well with the lack of Complex I in yeast (Yamashita *et al*., 2007). Complex II is regulated by SDHAF3 (also known as Lyrm10) and SDHAF1 (Lyrm8). These two proteins together mediate the transfer of Fe–S cluster to the SDHB subunit of complex II [37].

The FMC1 subfamily, though less prevalent, plays a crucial role as an assembly factor for Complex V [38,39]. It was suggested to stabilize ATP12p, an assembly factor for the F_1_ component of ATP synthase [38,40]. Importantly, FMC1 is the only LYRM that does not require the interaction with acylated ACP [8].

In addition, Lyrm5 regulates the activity of the electron-transferring flavoprotein (ETF) by removing its FAD cofactor [41]. ETF is a heterodimeric complex that accepts electrons from mitochondrial flavoenzymes and transfers them to the respiratory chain via ETF dehydrogenase [42].

The Lyrm1, Lyrm9 and MIEF1-MP subfamilies, though relatively less abundant, play crucial roles in the maturation of the mitoribosome. MIEF1-MP also known as AltMiD51 has been found as a part of the mitoribosome intermediate [43] where it stabilizes mitoribosome assembly factor MALSU1 [3]. Together these proteins possibly prevent premature association of large and small ribosome subunits [43]. Lyrm9, as predicted through bioinformatic analysis, interacts with the mitoribosome subunit MRPL57 and Lyrm1 [44]. Furthermore, Lyrm1 was recently predicted to interact with MTRF1L (mitochondrial translation release factor 1-like) by co-evolution analysis and structural modelling [44]. MTRF1L mediates the termination of the mitochondrial translation by recognizing two main termination codons [45].

Generally, most LYRM proteins maintain the characteristic LYR motif, typically followed by a downstream phenylalanine residue. However, certain adaptations in the protein sequence are observed in some LYRM subfamilies (electronic supplementary material, figure S2). Specifically, in the Lyrm5 subfamily, the arginine residue has been replaced by lysine, and in the Lyrm7 and FMC1 subfamilies, the leucine residue of the motif has been replaced by alanine or other hydrophobic amino acids. Notably, the FMC1 subfamily, lacking also the downstream phenylalanine, exhibits the most significant alteration from the classic LYRM signature sequence (electronic supplementary material, figure S2).

### 2.4. LYRMs are eukaryotic proteins that were fully represented in the LECA

The comparison of the LYRM’s presence across eukaryotes showed that all twelve subfamilies can be found in groups as diverse as animals and jakobids (figure 1*b*, electronic supplementary material, table S1) and that such distribution suggests their full presence in the LECA, which was a complex cell with all key eukaryotic traits [46]. According to the simplest scenario, the uneven distribution of LYRM proteins in individual eukaryotic lineages can thus be explained as independent losses of the ancestral state of the twelve LYRM proteins that were already present in the LECA together with ACP.

However, in contrast to the clear prokaryotic origin of ACP [6], so far there has been no record of the LYRM orthologue in prokaryotes [1]. Thus, it remained to be determined whether LYRMs could have been utilized by a mitochondrial ancestor [47,48] or an archaeal host [49,50] prior to the formation of the eukaryotic cell. We employed HMM-based searches, previously utilized for eukaryotic data, alongside the FoldSeek search engine to explore the presence of LYRM orthologues in bacterial and archaeal genome data (electronic supplementary material, table S3). Despite these efforts, neither approach was successful in identifying LYRM homologues in existing prokaryotes. This result strongly suggests their absence also in the organisms that gave rise to the eukaryotes. Consequently, this indicates that LYRMs are unique to eukaryotes, having been integrated into mitochondria to play a key role in controlling core functions such as the maturation of Fe–S proteins, the assembly of respiratory complexes and the mitoribosome.

### 2.5. Multifaceted interaction interface of LYRMs with the mitochondrial components of prokaryotic origin

We further examined the impact of functional diversification on the actual structure of a particular LYRM subfamily. Despite the sequence variability the experimental and modelled structures showed that the central triplet of α-helices is remarkably conserved across the subfamilies (figure 1*c*). Most LYRMs consist of solely the central triplet (electronic supplementary material, table S4), although additional structural features/domains could be previously identified in several LYRM sequences [1]. Given that the domain fusions may reflect the functional linkages [51] and hence provide additional information on the protein function, we analysed collected LYRMs for the presence of additional protein domains (electronic supplementary material, table S3). Interestingly, about 7% of the proteins were found to be associated with various known protein domains or fragments with unknown functions often with disordered regions. Of all the LYRM subfamilies, the most fusions and sequence extensions were identified in MIEF1-MP, Lyrm8, Lyrm7 and Lyrm4, with about 10% of the sequences involved.

**Figure 2.**
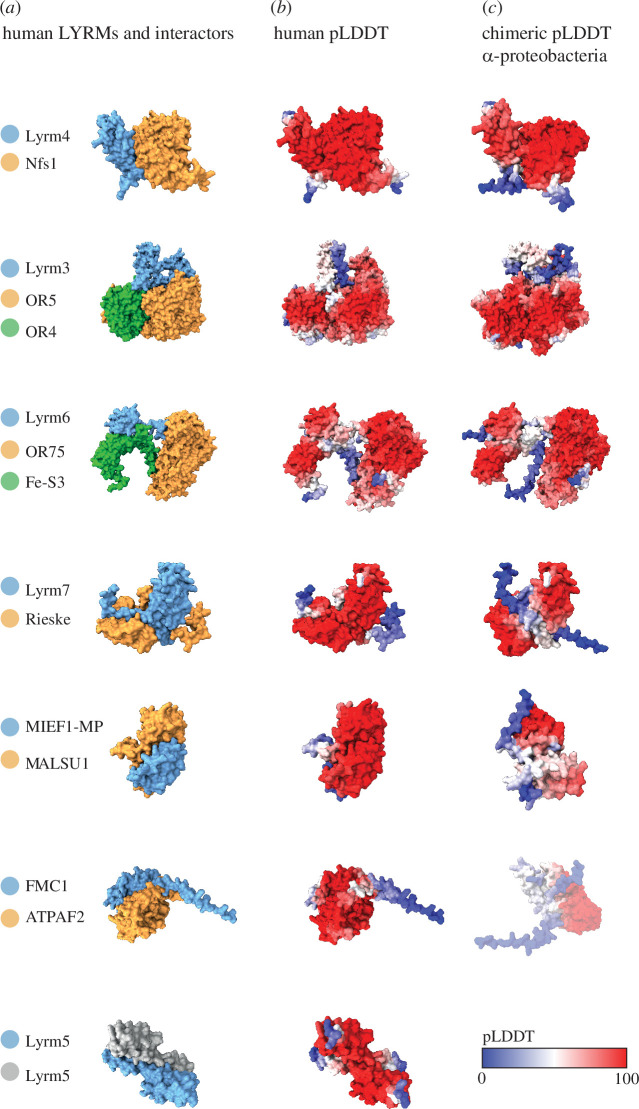
Multifaceted interaction of LYRMs with the mitochondrial components of prokaryotic origin. (*a*) Solved or predicted structures of human LYRMs when interacting with their mitochondrial targets coloured by subunits. (*b*) Human LYRMs in complexes coloured by pLDDT score. (*c*) Chimeric complexes of human LYRMs with the α-proteobacterial orthologues of the target proteins coloured by pLDDT score.

The LYRMs enter two different interactions: first, they all bind the same partner, acylated ACP, but at the same time each LYRM recognizes a different mitochondrial target protein. A key question we addressed was how such a structurally uniform protein family (figure 1*c*) could maintain specificity and avoid cross-reactions. We inspected the interactions of the LYRMs with their mitochondrial partners. At this stage, their interaction with ACP was not involved in the analysis. The available structural data (Lyrm3, Lyrm4, Lyrm6 and MIEF1) [35,43,52] or their predictions (FMC1) [44] were inspected to depict the contacts of α-helical triplet with the target protein. We also used available biochemical data [53] to predict the interaction of remaining LYRMs via Alphafold2. The accuracy of the approach was tested by modelling the interaction of Lyrm4 with Nfs1 and ACP, structures of which were already experimentally resolved [19]. Labelling the contact residues showed that the experimentally obtained results are nearly identical to our model (electronic supplementary material, figure S3). Through structural modelling, we confirmed the reported interaction between Lyrm7 and the Rieske protein [11,32] and predicted a potential dimerization of Lyrm5 via its extended third α-helix (figure 2*a,b*). The combined data showed that despite being a very uniform protein family, the LYRMs employ distinct regions of the α-helical triplet that enter the interaction with the target protein (figure 2). While this provides the specificity of the reaction, it also minimizes cross-reactivity among the subfamilies.

### 2.6. LYRMs control the function of original bacterial proteins in mitochondria

The interactions of the LYRMs with their cognate proteins stand at the centre of mitochondrial functions that are of bacterial origin [1]. Given that the respiratory complexes and mitochondrial ribosome underwent significant modifications when compared to their bacterial counterparts [54,55], we tested whether the LYRM interactors have clear bacterial orthologues or whether they represent such eukaryote-specific modifications that require eukaryote-specific LYRMs. Indeed, we found that all LYRM interactors have clear orthologues in bacteria (figure 2*c*; electronic supplementary material, figure S4). This suggests that in contrast to mitochondria, the orthologous bacterial complexes involved in respiration, translation and Fe–S cluster assembly do not require LYRMs for their function. Strikingly, a heterologous expression of yeast Lyrm4 in *E. coli* led to its complexing with the bacterial Nfs1 orthologue [56] suggesting that LYRMs could control the activity of the bacterial orthologues of their targets if co-existing in a single cell.

Whether the interaction could be theoretically possible for other functional pairs was tested by structural modelling of chimeric complexes consisting of the human LYRMs and the α-proteobacterial orthologues of their cognate targets (figure 2*c*). Interestingly, five out of six tested LYRMs were found to bind bacterial targets at the correct site, yet with a much lower pLDDT confidence score when compared to the natural complexes. No support was found for theoretical interaction between human FMC1 and bacterial ATP12 subunit. In general, the analysis suggested that the instalment of the LYRMs to the mitochondria was not accompanied by significant structural changes in their target proteins.

### 2.7. Acyl-independent interaction of FMC1 and the loss of hydrophobic channel of Lyrm4 in anaerobic eukaryotes

**Figure 3.**
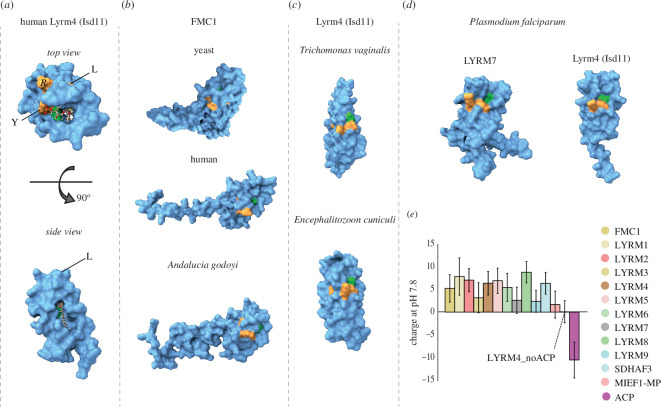
Evolutionary loss of acyl-ACP-dependent interaction with the LYRMs. (*a*) During the interaction of the LYRMs with the acylated ACP the acyl chain is inserted into the cavity formed by the central triplet of α-helices as illustrated by the modelled interaction of the acyl chain within human Lyrm4. LYR and F residues are highlighted in yellow and green, respectively. (*b*) Conserved absence of the cavity in FMC1 orthologues documented on yeast, human and *A. godoyi* proteins. (*c*) Lyrm4 orthologues from eukaryotes carrying MROs that lack electron transport chain and mtFAS. (*d)* Independent loss of the central cavity in the two LYRMs in the mitochondrion of *P. falciparum* and other apicomplexan parasites. (*e*) The total positive charge of the LYRMs diminishes in the ACP-lacking eukaryotes. The average charge of the proteins at pH 7.8 was calculated, and the error bars depict standard deviation as a measure of the value variations.

The function of the LYRMs is dependent on the interaction with the acylated ACP which inserts the acyl moiety into the hydrophobic tunnel formed by the tree α-helices of the LYRMs [19,52]. This interaction also requires the presence of the signature LYR motif [4,8,52] and the downstream conserved phenylalanine residue (LYR-F) [2,57] (figure 3*a*; electronic supplementary material, figure S2). While the LYRM-ACP complexes can form without the actual acylation of ACP, the lack of acylation ablates the function of the LYRMs [8]. The acylated ACP has thus been suggested to function as an allosteric activator of the LYRMs [8], yet the functional principle of this interaction remains largely unknown.

Yeast FMC1 is the only exception to the rule, as its function is not affected by the deletion of the LYR motif, nor does it require acylated ACP [8]. We thus examined if the predicted structure of yeast FMC1 explains the acyl-ACP independence. Indeed, the hydrophobic tunnel is absent not only in yeast FMC1 but also in FMC1 orthologues from other evolutionarily distant eukaryotes (e.g. *Andalucia godoyi*; figure 3*b*). Yet, modelling the FMC1-ACP complex showed that ACP is still capable of binding FMC1 (electronic supplementary material, figure S5) as also found previously by immunoprecipitation [8].

The conserved interaction of LYRMs with ACP could be documented by their co-occurrence in the vast majority of studied eukaryotes (figure 1*b*). Interestingly, some eukaryotes, which have adapted to anoxic environments including the extracellular and intracellular parasites, have lost ACP but retained LYRMs. In fact, in all identified cases, the loss of ACP is linked to the presence of a single LYRM subfamily, Lyrm4. Hence, we examined Lyrm4 orthologues from ACP-lacking eukaryotes (figure 3*c*), to see if the loss of interaction with the acylated ACP resulted in the disappearance of the hydrophobic tunnel and diminishing of the LYR-F motif. Indeed, the hydrophobic tunnel was found missing in all inspected proteins, similarly to FMC1. However, the LYR-F motif remained present with the phenylalanine directly neighbouring the tyrosine. While this nicely demonstrates the loss of allosteric regulation of these LYRMs by acylated-ACP, it raises a question of whether the LYR-F motif has perhaps other roles different from the interaction with ACP and the acyl ligand.

Additionally, we analysed two LYRMs in the mitochondrion of *Plasmodium falciparum*. Notably, *P. falciparum* and other apicomplexan parasites have lost the mFAS pathway but have retained the mitochondrial ACP. Interestingly, this ACP has lost its ability to attach an acyl chain owing to a mutation in a critical residue, yet it appears to still be capable of binding both *P. falciparum* LYRMs, as reported by Falekun *et al*. [58]. Like the anaerobic organisms, both Lyrm4 and Lyrm7 in *P. falciparum* lack the hydrophobic tunnel, despite preserving the LYR-F motif (figure 3*d*). This suggests that the mFAS pathway might have been rendered redundant in apicomplexan mitochondria owing to the unique existence of a similar pathway in their secondary plastid, the apicoplast [58]. This scenario thus nicely illustrates the distinct separation of two ACP roles: the regulation of LYRM protein activity and the synthesis of fatty acids.

Finally, we also examined the protein charges of both ACPs and LYRMs. ACPs are known to be acidic proteins with isoelectric points (pI) ranging from 3.9 to 4.3 [59], whereas LYRMs have been characterized as basic proteins [1]. This difference in charge between ACP and LYRMs leads to an intriguing hypothesis that their interaction might be driven by these opposing charges, facilitating the subsequent interaction of LYRMs with their specific partners. Consequently, we hypothesized that the absence of ACP in anaerobic organisms could result in a reduced charge in the remaining Lyrm4 proteins. Indeed, upon calculating the total charge for each LYRM subfamily, we observed that the charge of Lyrm4 proteins from anaerobic eukaryotes lacking ACP was significantly lower (0.09 at pH 7.8) compared to their aerobic counterparts, which had a charge of 6.4 (see figure 3*e*). However, it is important to note that our analysis of ‘anaerobic’ Lyrm4 proteins was based on a limited dataset of 21 proteins, in contrast to the much larger dataset of 2718 ‘aerobic’ counterparts.

## 3. Discussion

Recent studies have unveiled a surprisingly complex role of the LYRM proteins in mitochondrial metabolism [1–5]. They are involved in key mitochondrial processes, including respiration, translation and the formation of Fe–S clusters. Here, we show that twelve functionally specialized LYRMs are structurally related homologues, likely present in the LECA. This is evidenced by their presence in evolutionarily distant species such as animals and jakobids, the free-living protists with the largest mitochondrial genomes [60] and mitochondria rich in bacteria-like traits [61–63].

**Figure 4.**
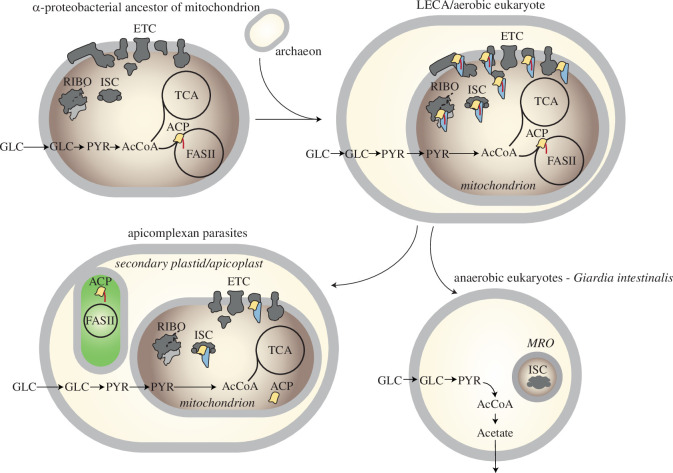
Hypothesis on the installation of the LYRM proteins to control the metabolism of the early mitochondrion. Based on the absence of the LYRMs in the extant prokaryotes, the bacterial ancestor of mitochondria, as well as the archaeal host, were probably devoid of the LYRM/ACP-dependent regulatory circuit. During the formation of the mitochondrion in the early eukaryotic cell, the LYRMs were introduced to control the function of pre-existing bacterial machineries of electron transport chain (ETC), Fe–S cluster assembly (ISC) and the mitoribosome (RIBO). The compartmentalization of the eukaryotic cell, pyruvate as the product of cytosolic glycolysis, has become transported to mitochondrion for its conversion into acetyl-CoA (AcCoA), a substrate of mitochondrial fatty acid synthesis (mtFAS/FASII) and tricarboxylic acid (TCA) cycle. Via the establishment of the LYRMs, the presence of acylated ACP has started to be used as a green light for the activation/assembly of ISC, ETC and the mitoribosome. However, during the course of evolution, some eukaryotes have reduced the role of the LYRMs in their mitochondria. In anaerobic eukaryotes, LYRMs repertoire became reduced to Lyrm4 or abandoned entirely whereas in apicomplexan parasites, mitochondrial adaptation led to the loss of mtFAS and ACP’s function has been limited to the interaction with the LYRMs.

The exact mechanism through which LYRMs modulate the functions of their target proteins remains largely unknown, but it is evident that their absence adversely affects the activity [5] or assembly [64] of these proteins. Consequently, LYRMs are acknowledged as key factors in assembly, activation or accessory subunits [1]. A notable aspect is the absence of LYRMs in bacteria, despite bacteria having orthologues of the same proteins that in mitochondria depend on LYRMs. Our study suggests that mitochondrial LYRMs might theoretically interact with bacterial orthologues of their targets, as exemplified by the experimental observation of Lyrm4 binding to the *E. coli* Nfs1 orthologue [56]. This observation prompts a crucial question regarding the role of LYRMs in mitochondrial evolution: specifically, what evolutionary advantages were gained by incorporating these small proteins into critical mitochondrial processes (figure 4).

A potential solution to this enigma lies in the mitochondrial fatty acid synthesis (mtFAS) pathway [65,66]. At the heart of mtFAS is the ACP, which serves as a scaffold for the elongation of fatty acyl chains. Initially, mtFAS was thought to be exclusively involved in the synthesis of lipoic acid. However, recent studies increasingly recognize ACP as a component of various mitochondrial protein complexes owing to its interaction with LYRMs [3]. An intriguing hypothesis suggests that the acylation of ACP may function as an indicator of mitochondrial acetyl-CoA levels [66] (figure 4). The presence of this central energy substrate and fatty acid precursor enables the formation of acylated-ACP through mtFAS. This acylated form of ACP, upon interacting with LYRMs, could then trigger the activation or assembly of Fe–S clusters, respiratory complexes and the mitoribosome [66]. Conversely, the absence of acetyl-CoA would inhibit the activation or assembly of these essential mitochondrial pathways.

The widespread and conserved occurrence of LYRMs and ACP across eukaryotes, ranging from free-living protists to multicellular organisms, indicates that this metabolic regulatory mechanism has been operational since the time of the LECA. On three occasions, evolution deviated from this blueprint. FMC1 does not require [8]—and, as shown in this work, cannot accommodate—acylated-ACP into its α-helical triplet, yet it can still interact with ACP alone. At increased temperatures FCM1 stabilizes ATP12p, an assembly factor for F_1_ ATP subcomplex [38]. F_o_F_1_ ATP synthase couples ATP synthesis with a transmembrane proton translocation but importantly, it can operate in reverse by coupling ATP hydrolysis with the reversed proton translocation in situations when the protein gradient is insufficient to support the import of proteins and other transport processes [67]. Thus, F_o_F_1_ ATP synthase must remain operational also in the absence of acetyl-CoA and the acylation independence of FMCO1 allows for continuing complex assembly in such conditions.

Apicomplexan parasites such as *P. falciparum* have lost the mtFAS pathway but have retained mitochondrial ACP (figure 4). Interestingly, this ACP still binds to Lyrm4 in its non-acylated form and contributes to Fe–S cluster assembly [58]. Similar to FMCO1, Lyrm4 and also here identified Lyrm7 orthologue of *P. falciparum* lost the space among the central triple of α-helices to accommodate the acyl chain on ACP. Loss of mtFAS in the apicomplexan protists that depend on aerobic respiration at least in part of their life cycles is intriguing and was perhaps possible owing to the existence of additional FASII pathway in the cell that is localized in the secondary plastid known as apicoplast [68].

Finally, as reported in this work, some anaerobes that reduced their mitochondrial metabolism to just the ISC pathway [18] retained only Lyrm4, which does not require interaction with acyl or ACP (figure 4). Some even abandoned Lyrm4 and their ISC pathway remained functional [69,70].

Interestingly, of the LYRM subfamilies, Lyrm4 represents the most conserved protein and is possibly ancestral to other LYRMs. Given that, the ISC pathway and the regulation of Nfs1 activity via acylated-ACP/Lyrm4 complex could be the initial process that employed LYRM-mediated control. Fe–S clusters are part of the respiratory complexes I, II and III and their formation via the ISC pathway is critical for the electron transport chain. Moreover, a recent report shows that Fe–S clusters are coordinated as structural features of the mitoribosome suggesting another vital link between the ISC pathway and the mitochondrial functions [71]. The successful introduction of the first LYRM protein within the ISC pathway might have paved the way for their expansion to other key sites of mitochondrial metabolism for tighter control.

Many aspects of LYRM/ACP-dependent regulation remain unknown such as the molecular basis for the LYRMs activation via acyl-ACP and parallel action on the wide range of client proteins. Were LYRMs originally introduced as mere adaptors of acylated ACP? In any case, LYRMs certainly mediate one of the most elegant metabolic regulatory circuits that may have played a key role in the evolution of the mitochondrion and its integration into the eukaryotic cell.

## 4. Methods

### 4.1. LYRMs identification

For the Lyrm/APC distribution in eukaryotes, we used the eukaryotic proteomes at UniProtKB release 2021_04 [72] and the EukProt database v. 3 [73]. Seed alignments of subsequent Pfam [74] families: Complex1_LYR (PF05347), Complex1_LYR_1 (PF13232) and Complex1_LYR_2 (PF13233) were used as queries for the individual searches. The HMMER [75] version 3.3 was used for the searches. For the ACP search, we first conducted a jackhmmer search against the EukProt. Obtained sequences were subsequently aligned using the Mafft v. 7.453 [76] with default parameters. The alignment was then used as a query for the HMM searches against selected proteomes [77].

The LYRM sequences from the 22 selected eukaryotic species were annotated using the InterProScan [78]. HHblits [79] was employed for cases where InterProScan proved insufficient to discern the specific subfamily. We annotated the proteins in accordance with the top hits with specific subfamily. Furthermore, we have made structure predictions for all the LYRMs from selected proteins using the AlphaFold2 [25] and used the models as queries for FoldSeek [27] searches against the AlphaFold structure database [25]. Since some of the subfamilies have additional structural features besides the central helical triplet (Lyrm3, Lyrm5, Lyrm7 and FMC1), they served as verification of annotations for those subfamilies.

### 4.2. Structural analysis

Both single-structure models and protein–protein complexes were obtained using the AlphaFold2 [25] Google Colab interface. The mmseqs2 option was used to build the MSAs. Additionally, we supplemented the AlphaFold2 with a custom MSA containing the sequences from the diverse and balanced dataset that was used for the phylogenetic analysis as it improved the pLDDT score of some models significantly. The ptm model was used for the single sequence predictions and the multimer_v2 for the complexes as the multimer_v3 proved to be unreliable often causing many clashes of the interacting proteins. The subsequent predictions of the ligand binding sites were done using the P2Rank v. 2.3.1 [80]. The hypothetical acyl binding sites were predicted employing the Autodock Vina v. 1.2.0 [81,82] using the 8Q1 acyl ligand from PDB. Structural alignments were performed using the DeepAlign [26] tools, namely, DeepAlign for the superposition of two proteins and 3DCOMB for multiple protein superpositions.

## Data Availability

All the data are part of the supplementary electronic files [83].
